# Cost-effectiveness of drug treatment for young and middle-aged stage 1 hypertensive patients with high risk

**DOI:** 10.7189/jogh.13.04147

**Published:** 2023-11-24

**Authors:** Yan-Feng Zhou, Hua Deng, Guo-Dong Wang, Shuohua Chen, Aijun Xing, Yanxiu Wang, Haiyan Zhao, Jingli Gao, Shouling Wu

**Affiliations:** 1 Department of Social Medicine, School of Public Health, Guangxi Medical University, Nanning, China; 2 Department of Nephrology, The First People's Hospital of Chenzhou, Chenzhou, China; 3 Department of Cardiology, Kailuan General Hospital, Tangshan, China; 4 Department of Intensive Care Unit, Kailuan General Hospital, Tangshan, China

## Abstract

**Background:**

Drug treatment was recommended for stage 1 hypertensive patients (blood pressure of 130-139 / 80-89 millimetres of mercury (mmHg)) with high cardiovascular disease (CVD) risk in the 2017 Hypertension Clinical Practice Guidelines, 2018 Chinese guidelines and 2021 World Health Organization guidelines, but not in other guidelines. However, evidence on the cost-effectiveness of drug treatment among young and middle-aged patients remains scarce. This study aimed to compare the cost-effectiveness of drug treatment vs. non-drug treatment for stage 1 hypertensive patients aged <60 years with high CVD risk.

**Methods:**

A microsimulation model projected quality-adjusted life years (QALYs), health care costs, and incremental cost-effectiveness ratios for drug treatment from a societal perspective. Transition probabilities were estimated from the Kailuan study with a sample size of 34 093 patients aged <60 years with high CVD risk. Costs and health utilities were obtained from the Kailuan study, national statistics reports and published literature.

**Results:**

Over a 15-year time horizon, the model predicted that drug treatment generated QALY of 9.36 and was associated with expected costs of 3735 US dollars ($) compared with 9.07 and $3923 produced by non-drug treatment among stage 1 hypertensive patients, resulting in a cost-saving for drug treatment. At a willingness-to-pay threshold of $10439/QALY (one gross domestic product (GDP) per capita in 2020), drug treatment had a 99.99% probability of being cost-effective for 10 000 samples of probabilistic sensitivity analysis. Sensitivity analyses by different values of transition probability, cost, utility and discount rate did not appreciably change the results. Shortening the time horizon to the average follow-up period of eight years resulted in ICER of $189/QALY for drug treatment (<1 × GDP/QALY).

**Conclusions:**

Our results suggested that drug treatment was a dominant strategy for stage 1 hypertensive patients aged <60 years with high CVD risk in China, which may provide evidence for policymakers and clinicians when weighing the pros and cons of drug treatment for young and middle-aged stage 1 hypertensive patients.

The definition of hypertension has been a hot topic of debate over the last few years. In 2017, the American College of Cardiology/American Heart Association (ACC/AHA) published new guidelines for treatment strategies for hypertension and stage 1 hypertension was defined as a systolic/diastolic blood pressure (SBP/DBP) of 130-139 / 80-89 millimetres of mercury (mm Hg) [1]. Conversely, the 2018 European Society of Cardiology/European Society of Hypertension (ESC/ESH) guidelines [2] and 2020 International Society of Hypertension (ISH) global hypertension practice guidelines [3] maintained the diagnostic threshold of hypertension at 140/90 mm Hg and recommended drug treatment for patients with clinical complications, e.g. cardiovascular disease (CVD) and diabetes mellitus, in the range of 130-139 / 80-89 mm Hg.

Recently, although individuals with SBP/DBP of 130-139 / 80-89 mm Hg were defined as non-hypertensive patients, the 2018 Chinese guidelines for the management of hypertension [4] and 2021 Guideline for the pharmacological treatment of hypertension in adults by the World Health Organization (WHO) [5] specially recommended drug treatment for those with high CVD risk (e.g. ≥3 CVD risk factors) and those who had pre-existing complications. Therefore, discrepancy existed for newly defined stage 1 hypertensive patients with high CVD risk, where drug treatment was recommended in the 2017 ACC/AHA guidelines [1], 2018 Chinese guidelines [4] and 2021 WHO guidelines [5], but not in 2018 ESC/ESH guidelines [2] and 2020 ISH guidelines [3].

Adopting new diagnostic criteria could significantly increase the prevalence of hypertension and substantially contribute to the high costs of health system. For example, the prevalence of hypertension was approximately doubled in most countries if the ACC/AHA guidelines were adopted [6,7] and an estimated additional $42.7 billion of direct medical cost would be required for lifetime therapy in China [8]. Therefore, it is critical to assess the cost-effectiveness of drug treatment among newly defined stage 1 hypertensive patients to provide evidence for the decision making of implementation of the new criteria in a “real world” practice, which will improve acceptance of national guidelines. We previously reported that drug treatment was not cost-effective compared to non-drug treatment for newly defined stage 1 hypertensive patients aged ≥65 years without CVD in China [9]. However, no such cost-effectiveness analysis has been conducted in young and middle-aged patients with high CVD risk. We hypothesised that drug treatment would be cost-effective among these young and middle-aged patients.

Here we aimed to determine the cost-effectiveness of drug treatment vs. non-drug treatment among stage 1 hypertensive patients aged <60 years with high CVD risk from a societal perspective using data from the Kailuan study.

## METHODS

### Study design and model population

We performed a model-based economic evaluation to assess the incremental cost-effectiveness ratio (ICER), with the outcomes expressed as cost per quality-adjusted life year (QALY) of drug treatment compared with non-drug treatment from a societal perspective for stage 1 hypertensive patients aged <60 years with high CVD risk. The ICER less than one time of per capita gross domestic product (GDP) per QALYs gained was considered as highly cost-effective [10]. According to the China Health Statistics Yearbook report, the per capita GDP was $10438.66 in 2020 (US$1.00 = 6.8974 Renminbi (RMB)) [11]. The model development and analyses were performed using TreeAge Pro Suite 2022 (TreeAge Software, Inc, Williamstown, Mass). This study conformed to the Consolidated Health Economic Evaluation Reporting Standards (CHEERS) guidelines and checklist items [12].

We modeled the cost-effectiveness of drug treatment for stage 1 hypertensive patients in the Kailuan study, a prospective dynamic cohort study in Tangshan, China. The detailed study design of the hypertension management programme has been described previously [13]. Briefly, the Kailuan study was initiated in 2006 and enrolled 101 517 employees in the 2006-2007 cycle and 25 337, 10 519, 21 651, 12 396, 7907 and 6921 new employees were additionally enrolled in 2008-2009, 2010-2011, 2012-2013, 2014-2015, 2016-2017 and 2018-2020 cycles, respectively. In the present study, we further excluded those with missing information of baseline BP (n = 2054), those with self-reported pre-existing CVD or diabetes at baseline (n = 16 304), those aged ≥60 years (n = 32 890), and those with less than three CVD risk factors (n = 91 344, defined as >55 years for men or >65 years for women, current smoking, low-density lipoprotein cholesterol ≥3.4 millimoles per litre (mmol/l) (130 milligrammes per decilitre (mg/dl)), high-density lipoprotein cholesterol <1.04 mmol/l (40 mg/dl), fasting blood glucose >6.0 mmol/l or body mass index (BMI)≥28.0 kilogrammes per square metre (kg/m^2^), according to the 2018 Chinese guidelines) [4], those with optimal BP or elevated BP levels (n = 9563, defined as SBP<130 mm Hg and DBP<80 mm Hg), leaving 34 093 participants in the study population. A flowchart of sample selection is shown in Figure S1 in the **Online Supplementary Document**, and baseline characteristics of study participants are shown in Table S1 in the **Online Supplementary Document**.

### Model overview

**Figure 1:**
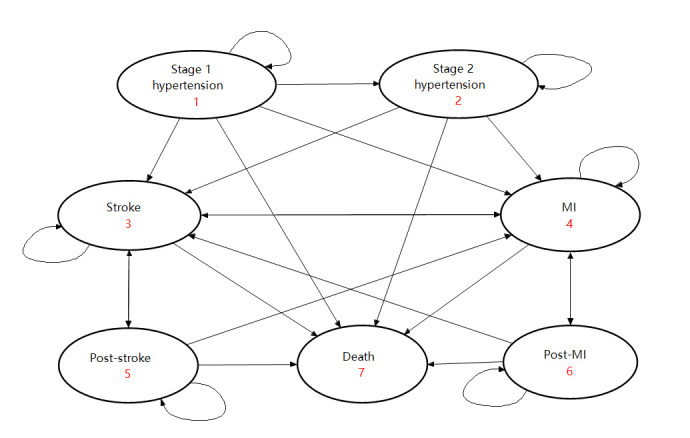
Markov state transition model for stage 1 hypertension. MI –myocardial infarction.

Based on previously published models [9], a Markov microsimulation model was constructed to simulate hypertension progression. We simulated two strategies for the study population: one receiving drug treatment and the other receiving non-drug treatment. We modeled the following distinct health states: (1) stage 1 hypertension, defined as SBP/DBP of 130-139/80-89 mm Hg without antihypertensive medications; (2) stage 2 hypertension, defined as SBP/DBP≥140/90 mm Hg or having been clinically diagnosed with hypertension or having received any antihypertensive medications; (3) stroke; (4) myocardial infarction (MI); (5) post-stroke; (6) post-MI; (7) death. Patients with stage 1 hypertension can maintain the disease status, progress to stage 2 hypertension, suffer a stroke/MI, or die directly. Stroke/MI patients may move to a chronic health state (post-stroke/post-MI), suffer a recurrent stroke/MI, be complicated by MI/stroke, or die directly (**Figure 1**).

As the average length of follow-up of the Kailuan study population included in this analysis was 8.23 years (range = 1.00 to 14.56 years), we simulated an eight-year time and 15-year time horizon to quantify potential benefits of drug treatment for those of young and middle-aged patients. The model cycle length was 1 year, and a half-cycle correction was applied.

For transition probability, all-cause mortality and event rates for stroke and MI among stage 1 and stage 2 hypertensive patients were derived from the Kailuan study. In the Kailuan study, person-years for each participant were counted from the baseline survey until the date of death or 31 December 2020, whichever came first. The database of CVD diagnoses was obtained from the Municipal Social Insurance Institution and Hospital Discharge Register and was updated annually during the follow-up periods. All-cause mortality was linked to the municipal death registries and checked annually against local residential records, with active confirmation of survival through subdistrict offices. We used ICD-10 codes to identify CVD cases (I21 for MI, I60, I61 and I63 for stroke) [13]. The recurrence rates of stroke and MI were obtained from the Hospital Discharge Register until 13 December 2020. The frequency of progression to stage 2 hypertension in stage 1 hypertensive participants was calculated from the baseline survey until the first onset of stage 2 hypertension. Relative risk (RR) reductions on first events, recurrence events, and postponing hypertension progression were derived from published literature [14-18]. Event rates are summarised in **Table 1**.

**Table 1:** Selected input value for the cost-effectiveness model

Data input	Stage 1 hypertensive patients aged <60 years with high cardiovascular risk*	Stage 2 hypertensive patients aged <60 years with high cardiovascular risk*
**First cardiovascular disease event or death, rate per 1000 person-year (95% CI)**		
Stroke incidence	2.9 (2.6-3.2)	8.1 (7.7-8.5)
MI incidence	1.3 (1.1-1.5)	2.3 (2.1-2.5)
All-cause mortality	4.1 (3.7-4.5)	8.9 (8.2-9.1)
**Second cardiovascular disease event or death, rate per 1000 person-year (95% CI)**		
MI incidence after stroke	0.03 (0.01-0.1)	0.13 (0.08-0.20)
Recurrent stroke	31.1 (22.5-42.9)	55.1 (49.1-61.9)
All-cause mortality after stroke	0.3 (0.2-0.5)	1.4 (1.2-1.6)
Stroke incidence after MI	0.02 (0.01-0.08)	0.09 (0.06-0.2)
Recurrent MI	36.4 (23.7-55.8)	44.9 (36.2-55.7)
All-cause mortality after MI	0.2 (0.1-0.3)	0.4 (0.3-0.5)
Frequency of progression to stage 2 hypertension, year, %	24.08 (21.72-25.52)	-
**Effect of drug treatment**		
RR for risk of stroke [14]	0.85 (0.68-1.06)	0.86 (0.72-1.01)
RR for risk of recurrent stroke [14,15]	0.68 (0.56-0.84)	0.74 (0.67-0.81)
RR for risk of MI [14]	0.98 (0.88-1.09)	0.86 (0.76-0.96)
RR for risk of recurrent MI [15]	0.73 (0.64-0.82)	0.68 (0.58-0.80)
RR for risk of all-cause mortality [14]	0.98 (0.90-1.06)	0.87 (0.75-1.00)
RR for risk of hypertension progression [16, 17]	0.61 (0.53-0.70)	NA
**Cost,** $†		
Annual cost for hypertension screening or management [19]	28.71 (±25%)	
Annual drug cost of 1.0 standard dose [20]	88.92 (±25%)	
Annual cost of productivity loss	275.38 (±25%) in the non-drug treatment, 4.8% reduction in the drug treatment	
Annual cost for stroke [9,11]	3249.55 (±25%) for the first year, 1525.64 (±25%) for the subsequent years	
Annual cost for MI [9,11]	4710.83 (±25%) for the first year, 428.26 (±25%) for the subsequent years	
**Quality of life weights (health utilities)**		
Hypertension [9]	0.90 (0.79-0.95)	
Stroke [9]	0.63 (0.26-0.88); post 0.65 (0.46-0.82)	
MI [9]	0.76 (0.50-0.89); post 0.88 (0.67-0.94)	
Death	0	
**Discount rate**	5% (0%-8%)	

CI – confidence interval, MI – myocardial infarction, NA – not applicable, $ – US dollar, RMB – Chinese renminbi

*The values inside the brackets represent ranges for sensitivity analyses.

†The costs were inflated to the 2020 price level and converted to US dollars (US$1.00 = 6.8974RMB).

### Costs

The cost-effectiveness analyses were performed from a societal perspective and therefore both direct and indirect costs were included. These costs included hypertension screening or management costs [19], antihypertensive drug costs, productivity losses, and costs for stroke and MI. Average antihypertensive drug costs of 1.0 standard dose per year were calculated using the annual cost of 62 antihypertensive medications and the prescription frequency for each medication [20]. Productivity losses resulting from early retirement due to ill health were obtained through the Social Security System. Due to a lack of information, we did not consider other productivity losses, e.g. absenteeism. According to the provisional regulations about retirement and resignation of workers approved by the State Council, the retirement age was 60 years for men and 55 years for women [21]. Early retirement due to ill health can be defined as the full exit from an organisational job or career path of a long duration before mandatory retirement age due to illness [22]. The length of early retirement due to ill health was calculated by the legal retirement age minus the actual retirement age. Productivity loss was calculated by the length of early retirement times the average wage. A lower or higher estimate of productivity loss was calculated using a reduced or an additional 25% of the average wage rate, respectively. As the mean age was 45.9 years and the average cumulative length of early retirement was 9.5 years for the study population, productivity losses were only considered in the first 10 years of the microsimulation.

Annual hospitalised costs for stroke, MI, and post-stroke/post-MI were extracted from the electronic medical records in the Kailuan study and checked through the China Health Statistics Yearbook 2020 and published literature [9,11]. The costs were inflated to the 2020 price level using the average inflation rate in China and converted to US dollars. Cost details are summarised in **Table 1**. All costs were discounted at 5% annually. For the sensitivity analyses, we used standard errors from the literature to define probability distributions for costs. If these were not available, we assumed the standard error was ±25% of the mean [23].

### QALY and health utilities

Utility values of hypertension, stroke, MI, post-stroke, and post-MI were obtained from published literature based on the European Quality of Life-5 Dimensions Questionnaire (EQ-5D) (**Table 1**) [9]. QALY was calculated by multiplying the time duration in a certain health state by the utility value associated with that state. The QALYs after one year were discounted at an annual rate of 5%.

### Cost-effectiveness analysis

The estimated values of model parameters combined with the assumptions of costs and effectiveness made above were used to calculate the ICER between drug treatment and non-drug treatment over a 15-year time horizon and 8-year time horizon.

### Scenario and sensitivity analyses

The impact of uncertainty around the model’s parameters on the cost-effectiveness results was assessed using scenario and sensitivity analyses. Specifically, scenario analyses were used to explore the sensitivity of results to alternative model assumptions and parameter values chosen. For instance, as the mean (standard deviation (SD)) age was 45.9 (11.0) years for the model population (Table S1 in the **Online Supplementary Document**), we simulated a longer time horizon of 55 years, by which time most people in the model would have died (to age 100). In addition, productivity losses were further excluded from the model to explore the robustness of the results [24].

One-way and probabilistic sensitivity analyses (PSA) were performed to determine how key model parameters would affect the results. First, we conducted one-way sensitivity analyses, including different values of transition probability, cost, utility, and discount rate. Second, to assess how sensitive the results were to variations in simultaneous changes of several variables, we conducted a PSA with a set of 10 000 produced results characterised by the probability distributions of outcomes resulting from the uncertainty around the input parameters. To quantify the uncertainty captured by the sensitivity, we used 10 000 Monte Carlo simulations to perform *t* tests and establish whether the costs and effectiveness associated with drug treatment and non-drug treatment were statistically different from one another [25]. Two-sided *P* < 0.05 was considered as statistical significance. We assumed beta distributions for clinical events rates, transition probabilities, and health utilities; a gamma distribution for costs (Table S2 in the **Online Supplementary Document**). In addition, the cost-effectiveness acceptability curve was constructed to assess the probability of cost-effectiveness at a willingness-to-pay (WTP) threshold of 1 to 3 times per capita GDP ($10 439 to $31 316).

## RESULTS

### Main analysis

The model predicted that drug treatment generated QALY of 9.36 and was associated with expected costs of $3735 in comparison with 9.07 and $3923 produced by non-drug treatment over a 15-year time horizon (**Table 2**). Although the costs for stage 1 hypertension were higher in drug treatment group than in non-drug treatment group ($709.74 vs. $85.87), the costs for stage 2 hypertension, stroke, MI, and productivity loss were lower in drug treatment group than in non-drug treatment group (total $3025.29 vs. $3837.60; **Table 2**), which resulted in a cost-saving for drug treatment. In health economics, dominant means a specific scenario when cost is lower and health outcome is better [26]. As such, drug treatment dominates non-drug treatment over a 15-year time horizon.

**Table 2:** Results for drug treatment vs. non-drug treatment among stage 1 hypertensive patients aged <60 years with high cardiovascular risk

Outcome	8-year time horizon			15-year time horizon		
**Drug treatment**	**Non-drug treatment**	**Changes**	**Drug treatment**	**Non-drug treatment**	**Changes**
Discounted QALYs	5.92	5.80	0.12	9.36	9.07	0.29
Discounted cost ($)*	2603.38	2582.13	21.25	3735.03	3923.47	-188.44
Costs by stage 1 hypertension†	552.54	80.52	472.02	709.74	85.87	623.87
Costs by stage 2 hypertension	215.95	414.38	-198.43	493.39	788.8	-295.41
Costs by stroke	89.08	237.99	-148.91	337.68	701.24	-363.56
Costs by MI	19.24	67.43	-48.19	59.30	148.75	-89.45
Productivity loss**‡**	1726.57	1781.81	-55.24	2134.92	2198.81	-63.89
Discount ICER	$188.50/QALY			Cost-saving		

QALY – quality-adjusted life-year, $ – US dollar, MI – myocardial infarction, ICER – incremental cost-effectiveness ratio

*US$1.00 = 6.8974 renminbi (RMB). The per capita gross domestic product (GDP) of China in 2020 was reported to be $10438.66 from the China Health Statistics Yearbook report.

†Costs for stage 1 hypertension in drug treatment group included costs for hypertension screening or management and costs for antihypertensive drugs, whereas costs for stage 1 hypertension in non-drug treatment group only included costs for hypertension screening or management.

‡Productivity losses were only considered in the first 10 years of the microsimulation.

When shorted the time horizon to 8 years while holding all other base-case assumptions, the model predicted that drug treatment generated QALY of 5.92 and was associated with expected costs of $2603 in comparison with 5.80 and $2582 produced by non-drug treatment, which resulted in an ICER of $189/QALY for drug treatment (<1 × GDP/QALY, **Table 2**).

### Scenario and sensitivity analyses

The results of scenario analyses show that drug treatment generated QALY of 15.68 and was associated with expected costs of $6350 in comparison with 14.93 and $7062 produced by non-drug treatment, resulting in cost-saving or dominant for drug treatment compared with non-drug treatment over a 55-year horizon (Table S3 in the **Online Supplementary Document**). In addition, the exclusion of productivity loss showed consistent results with the main results (Table S4 in the **Online Supplementary Document**).

The results of one-way sensitivity analyses are shown in Figure S2 and Table S5 in the **Online Supplementary Document**. Sensitivity analysis of transition probability, cost, utility parameters, and discount rate did not change the ranking of the ICERs. The top three parameters that had potential impacts on the ICERs were cost attributable to productivity loss, RR reduction in stroke and mortality with antihypertensive therapy.

*T* tests for means performed using the 10 000 Monte Carlo simulations values provided the following results: 1) costs of drug treatment and non-drug treatment are statistically different over 15-year time horizon with *P* value <0.001; the mean cost of drug treatment and non-drug treatment is $3734 (SD = $586) and $3929 (SD = $588), respectively; 2) the effectiveness of drug treatment and non-drug treatment are statistically different with *P* value <0.001; the mean effectiveness of drug treatment and non-drug treatment is 9.36 (SD = 0.80) and 9.07 (SD = 0.76), respectively.

**Figure 2:**
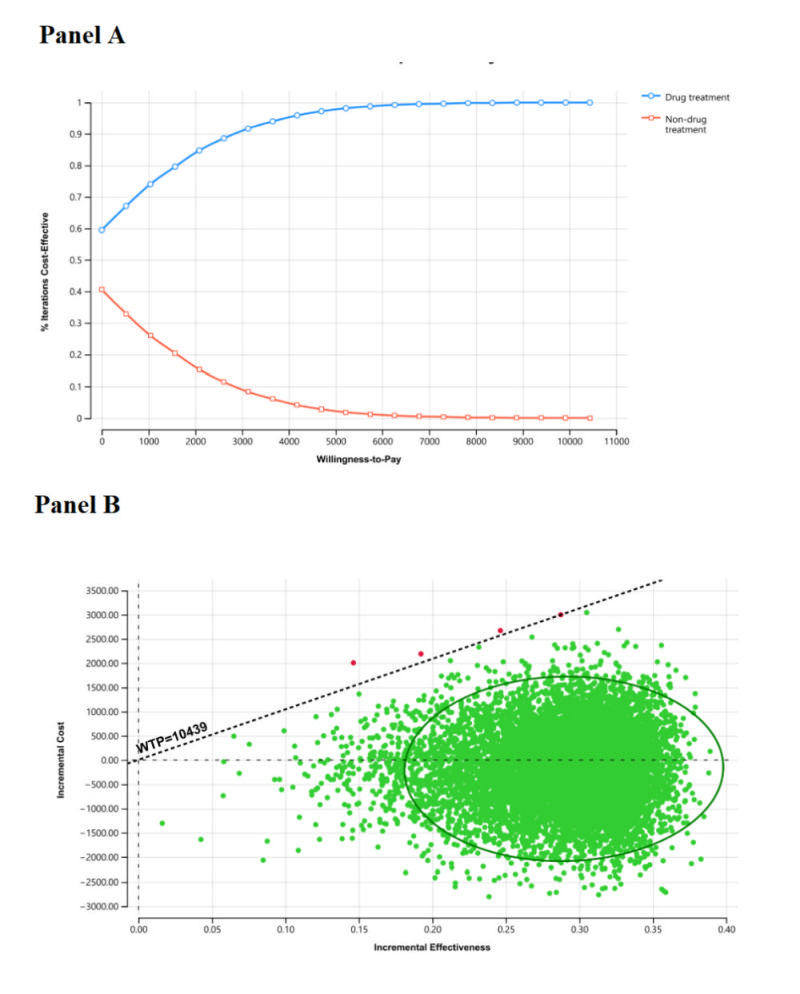
Cost-effectiveness acceptability curve and incremental cost-effectiveness scatter plots of drug treatment for young and middle-aged stage 1 hypertensive patients with high cardiovascular risk. **Panel A.** Cost-effectiveness acceptability curve. **Panel B.** Incremental cost-effectiveness scatter plots-WTP = $10 439/QALY (1 GDP/QALY). QALY – quality-adjusted life year, GDP – gross domestic product, WTP – willingness-to-pay

**Figure 2** and Figure S3 in the **Online Supplementary Document** present the cost-effectiveness acceptability curve (**Figure 2**, panel A) and cost-effectiveness scatter plots (**Figure 2**, panel B). At a WTP threshold of $10 439/QALY, drug treatment had a 99.99% probability of being cost-effective over a 15-year time horizon. At a WTP threshold of $31 316/QALY, drug treatment had a 100% probability of being cost-effective over a 15-year time horizon.

## DISCUSSION

To our knowledge, this is the first study in the Chinese population investigating the cost-effectiveness of drug treatment vs. non-drug treatment in young and middle-aged stage 1 hypertensive patients using local incidence events and cost data. The model-based economic analysis indicated that drug treatment generated less costs from stage 2 hypertension, CVD, and productivity loss, but was more effective than non-drug treatment, suggesting that drug treatment was a dominant strategy for stage 1 hypertensive patients aged <60 years with high CVD risk over a 15-year time horizon. In addition, modeling results were robust with respect to a comprehensive sensitivity analysis.

Prior studies have suggested that treatment for patients with SBP/DBP≥140/90 mm Hg was cost-effective or cost-saving, regardless of CVD risk [27,28]. In addition, multiple studies have found that intensive hypertension control (target, SBP<120 mm Hg or SBP<133 mm Hg) was cost-effective compared with standard hypertension control (target, SBP<140 mm Hg) [29,30]. Collectively, previous studies have shown that controlling hypertension was cost-effective and intensive control appeared to provide the most value. In addition to drug treatment, lifestyle modifications remain an important component for BP control.

Nevertheless, the cost-effectiveness of drug treatment for individuals with SBP/DBP of 130-139 / 80-89 mm Hg has not been extensively examined. Using data from a cross-sectional study and published literature, Chen and colleagues [31] found that drug treatment was cost-effective for prehypertension patients (130-139 / 85-89 mm Hg) compared with placebo treatment, with an ICER of $12 994/QALY over a lifetime. However, they did not limit the study population to high CVD risk, whereas drug treatment was only recommended for individuals with high or very high CVD risk in recent hypertension guidelines [1,2,4]. In addition, due to lacking relevant data, they made the conservative assumption of no effect on CVD risk by drug treatment and did not consider any cost of productivity loss. Conversely, our previous study reported that drug treatment was not cost-effective for stage 1 hypertensive patients aged ≥65 years without CVD [9]. The explanation for the inconsistency may be that treatment effects were greater in young individuals than in older individuals [32]. In addition, productivity loss due to illness may be more pronounced among young and middle-aged individuals.

In our study, drug treatment entailed additional medication use, thus the costs for stage 1 hypertension were higher in the drug treatment than in non-drug treatment. However, these costs were offset by lower costs from stage 2 hypertension, CVD and productivity loss, resulting in less total costs and cost savings. In terms of effectiveness, a recent systematic review and meta-analysis of 74 unique trials showed that drug treatment might not be associated with reduced risk of CVD and mortality at lower BP levels (SBP<140 mm Hg) [14]. However, included studies were not limited to patients with high CVD risk. In addition, treatment of stage 1 hypertension could significantly reduce the risk of progression to manifest stage 2 hypertension [16,17], thus, in turn, reducing the risk of subsequent CVD. Taken together, in a microsimulation modeling study, we further indicated that drug treatment for stage 1 hypertension with high CVD risk might add substantial value; however, treatment effects for patients with relatively low CVD risk were largely unknown and required further exploration.

Our findings have several public health implications. Despite the 2017 ACC/AHA guidelines would result in a substantial increase in the proportion of patients labeled as having hypertension, there might be a slight increase in the percentage of patients who are recommended drug treatment by using a combination of BP levels and CVD risk [1]. For example, these patients represent only 1.9% of US adults [33] and 2.0% of the general Chinese population [34]. Furthermore, young adulthood and middle age are critical periods for chronic disease prevention, when individuals are at their highest productivity [35]. Illness at a young age may result in the loss of productivity at late life, whereas early treatment of hypertension may retard BP progression and prevent resistant hypertension and complications [34]. Taken together, the increase in the number of high-risk patients requiring treatment would be marginal according to the 2017 guidelines, whereas early detection and treatment of young and middle-aged patients could be cost-effective ways to improve BP control and reduce CVD-related events, and finally foster improvements in individual health and organisational productivity.

### Study limitations

Several limitations need to be acknowledged. First, our model only captured a limited number of health states, which might have underestimated the benefits of drug treatment for newly defined stage 1 hypertensive patients. Second, the recurrence rates and RR reductions were derived from published literature, in which some participants were not at a high risk of CVD. As expected, the RR reductions were greater in participants with high CVD risk compared to those with low CVD risk, resulting in underestimating QALYs and ICERs for drug treatment. Third, no robust data was available for medication adherence, so its potential impact on our results might deserve more attention. However, the potential impact of suboptimal medication adherence was likely to be mitigated by the conservative assumptions already described [36]. Fourth, lifestyle changes were estimated to have potentially significant impacts on cardiovascular morbidity and mortality [4]. However, there was insufficient data to model the effect of lifestyle changes in drug and non-drug groups; we then assumed that there would be little changes in the two groups and the non-drug is the natural disease progression. Thus, our results should be considered hypothesis-generating. Fifth, although the study was undertaken in a “real world” practice and was more representative of reality, using a single study as a vehicle for economic analysis is still an inadequate and partial basis for decision-making. Furthermore, our study was conducted in an Asia population and most participants were men; hence the findings could be dependent on the local effect which limits generalisability. More studies are still needed to confirm our findings and reach a consensus.

## CONCLUSIONS

The results of the present study confirmed the hypothesis that drug treatment was cost-effective for stage I hypertensive patients aged <60 years with high CVD risk over a 15-year time horizon, as it proved lower costs from stage 2 hypertension, CVD, and productivity loss, and resulted in less total costs and cost savings. More studies are still needed to validate our results and provide more evidence for policymakers and clinicians when weighing the pros and cons of drug treatment for young and middle-aged stage 1 hypertensive patients.

## Additional material


Online Supplementary Document
