# Supplementary material for: Cost-effectiveness of drug treatment for young and middle-aged stage 1 hypertensive patients with high risk
Source: J Glob Health. 2023 Nov 24;13:04147. doi: 10.7189/jogh.13.04147 (PMC10668205; doi:10.7189/jogh.13.04147)
Supplement: Online Supplementary Document [file jogh-13-04147-s001.pdf]

Cost-effectiveness of drug treatment for young and middle-aged stage 1 hypertensive patients with high risk

Supplemental Materials

Table S1. Baseline characteristics of study participants

Table S2. Distributions of input parameters for probabilistic sensitivity analysis

Table S3. Scenario analysis by simulating the model for 55-year time horizon

Table S4. Scenario analysis by excluding cost of productivity loss

Table S5. Tornado text report (ICER), drug treatment versus non-drug

Figure S1. Participant flow chart

Figure S2. Sensitivity analysis of model variables

Figure S3. The cost-effectiveness scatter plots for drug treatment versus non-drug treatment

Table S1. Baseline characteristics of study participants

Variables	Stage 1 hypertension without drug treatment (N=12500)	Stage 2 hypertension (N=21593)
Age, mean (SD), y	45.89 (11.01)	50.77 (8.40)
BMI, mean (SD), kg/m^2	26.48 (3.62)	27.15 (3.57)
Systolic BP, mean (SD), mm Hg	124.8 (8.3)	147.0 (16.6)
Diastolic BP, mean (SD), mm Hg	81.7 (3.7)	94.2 (9.9)
TC, mean (SD), mmol/L	5.74 (1.43)	5.76 (1.38)
LDL-C, mean (SD), mmol/L	3.29 (1.14)	3.23 (1.28)
HDL, mean (SD), mmol/L	1.38 (0.47)	1.44 (0.50)
FBG, mean (SD), mmol/L	5.47 (0.76)	5.62 (0.76)
Men	11750 (94.00)	20177 (93.44)
Physically active *	2073 (16.58)	3827 (17.72)
Current smoker	8604 (68.83)	13235 (61.29)

BP indicates blood pressure; BMI, body mass index; FBG, fasting blood glucose; HDL-C, high-density lipoprotein cholesterol; LDL-C, low-density lipoprotein cholesterol; TC, total cholesterol.

* Being physically active was defined as moderate or vigorous physical activity for ≥80 minutes per week.

Table S2. Distributions of input parameters for probabilistic sensitivity analysis

Dist–Utility for Hypertension [1-3]	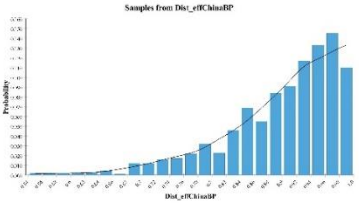	Source: Pan, 2018; Zhang, 2016; Geisler, 2012<br>Beta, Real-numbered parameters, alpha = (((0.90)^2)*(1-(0.90))/((0.08)^2)-(0.90)), beta = ((1-(0.90))*(((1-(0.90))*(0.90))/((0.08)^2)-1)); Expected value: 0.90
Dist–Utility for stroke [2, 3]	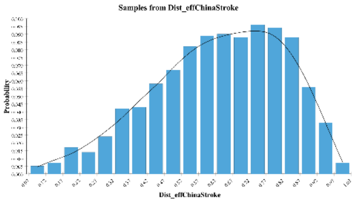	Source: Pan, 2018; Geisler, 2012<br>Beta, Real-numbered parameters, alpha = (((0.63)^2)*(1-(0.63))/((0.19)^2)-(0.63)), beta = ((1-(0.63))*(((1-(0.63))*(0.63))/((0.19)^2)-1)); Expected value: 0.63
Dist–Utility for post-stroke [4]	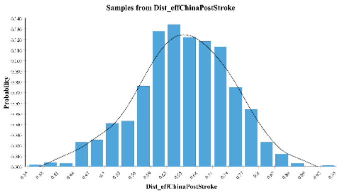	Source: Bress, 2017<br>Beta, Real-numbered parameters, alpha = (((0.65)^2)*(1-(0.65))/((0.09)^2)-(0.65)), beta = ((1-(0.65))*(((1-(0.65))*(0.65))/((0.09)^2)-1)); Expected value: 0.65
Dist–Utility for MI [3, 5]	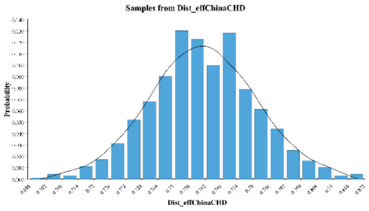	Source: Geisler, 2012; Wang, 2015<br>Beta, Real-numbered parameters, alpha = (((0.76)^2)*(1-(0.76))/((0.02)^2)-(0.76)), beta = ((1-(0.76))*(((1-(0.76))*(0.76))/((0.02)^2)-1)); Expected value: 0.76
Dist–Utility for post-MI [3]	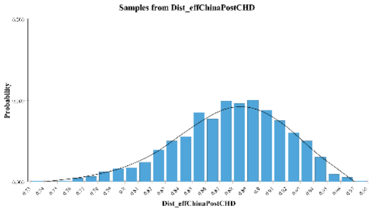	Source: Geisler, 2012<br>Beta, Real-numbered parameters, alpha = (((0.88)^2)*(1-(0.88))/((0.04)^2)-(0.88)), beta = ((1- ((1-(0.88))*(((1-(0.88))*(0.88))/((0.04)^2)-1)); Expected value: 0.88
Dist-hypertension screening or checkups	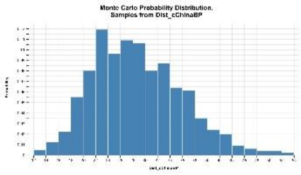	Source: Kailuan study<br>Gamma, alpha = ((28.71)^2)/((7.18)^2), lambda = (28.71)/((7.18)^2); Expected value: 28.71
Dist–Cost for antihypertensive drug [6, 7]	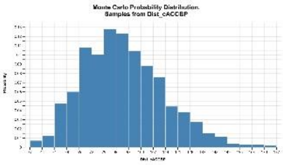	Source: Zhou, 2021; Su, 2017<br>Gamma, alpha = ((88.92)^2)/((22.23)^2), lambda = (88.92)/((22.23)^2); Expected value: 88.92

Dist–Cost for productivity loss in drug treatment group	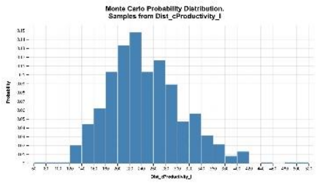	Source: Kailuan study<br>Gamma, alpha = ((262.16)^2)/((65.54)^2), lambda = (262.16)/((65.54)^2); Expected value: 262.16
Dist–Cost for productivity loss in non-drug treatment group	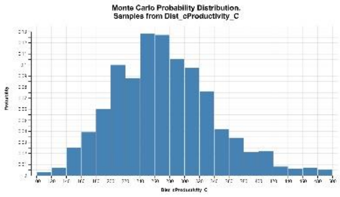	Source: Kailuan study<br>Gamma, alpha = ((275.38)^2)/((68.85)^2), lambda = (275.38)/((68.85)^2); Expected value: 275.38
Dist–Cost for stroke year 1 (annually) [8, 9]	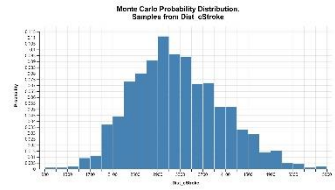	Source: Chen, 2017; The Ministry of Health of China, 2019; Zhou, 2021<br>Gamma, alpha = ((3249.55^2)/((812.39)^2), lambda = (3249.55)/((812.39)^2); Expected value: 3249.55
Dist–Cost for post stroke yrs. 2+ (annually) [9]	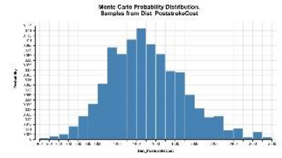	Source: Zhou, 2021<br>Gamma, alpha = ((1525.64)^2)/((381.41)^2), lambda = (1525.64)/((381.41)^2); Expected value: 1525.64
Dist–Cost for MI year 1 (annually) [8, 9]	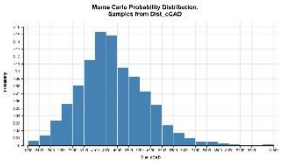	Source: The Ministry of Health of China, 2019; Zhou, 2021<br>Gamma, alpha = ((4710.83)^2)/((1171.71)^2), lambda = (4710.83)/((1171.71)^2); Expected value: 4710.83
Dist–Cost for post MI yrs. 2+ (annually) [9]	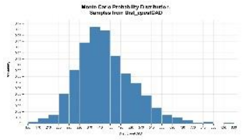	Source: Zhou, 2021<br>Gamma, alpha = ((428.26)^2)/((107.07)^2), lambda = (428.26)/((107.07)^2); Expected value: 428.26

MI indicates myocardial infarction; and RR, relative risk.

References

1. Zhang Y, Zhou Z, Gao J, Wang D, Zhang Q, Zhou Z, Su M, Li D: **Health-related quality of life and its influencing factors for patients with hypertension: evidence from the urban and rural areas of Shaanxi Province, China**. *BMC Health Serv Res* 2016, **16**:277.
2. Pan CW, Cong XL, Zhou HJ, Wang XZ, Sun HP, Xu Y, Wang P: **Evaluating health-related quality of life impact of chronic conditions among older adults from a rural town in Suzhou, China**. *Arch Gerontol Geriatr* 2018, **76**:6-11.

3. Geisler BP, Egan BM, Cohen JT, Garner AM, Akehurst RL, Esler MD, Pietzsch JB: **Cost-effectiveness and clinical effectiveness of catheter-based renal denervation for resistant hypertension**. *Journal of the American College of Cardiology* 2012, **60**(14):1271-1277.
4. Bress AP, Bellows BK, King JB, Hess R, Beddhu S, Zhang Z, Berlowitz DR, Conroy MB, Fine L, Oparil S *et al*: **Cost-Effectiveness of Intensive versus Standard Blood-Pressure Control**. *The New England journal of medicine* 2017, **377**(8):745-755.
5. Wang L, Wu YQ, Tang X, Li N, He L, Cao Y, Chen DF, Hu YH: **Profile and Correlates of Health-related Quality of Life in Chinese Patients with Coronary Heart Disease**. *Chin Med J (Engl)* 2015, **128**(14):1853-1861.
6. Zhou YF, Liu N, Wang P, Jeong Yang J, Song XY, Pan XF, Zhang X, He M, Li H, Gao YT *et al*: **Cost-Effectiveness of Drug Treatment for Chinese Patients With Stage I Hypertension According to the 2017 Hypertension Clinical Practice Guidelines**. *Hypertension* 2020, **76**(3):750-758.
7. Su M, Zhang Q, Bai X, Wu C, Li Y, Mossialos E, Mensah GA, Masoudi FA, Lu J, Li X *et al*: **Availability, cost, and prescription patterns of antihypertensive medications in primary health care in China: a nationwide cross-sectional survey**. *Lancet* 2017, **390**(10112):2559-2568.
8. China TMoHo: **China's health and family planning statistical yearbook 2019**. Beijing: China Union Medical University; 2019.
9. Zhou YF, Chen S, Wang G, Chen S, Zhang YB, Chen JX, Tu ZZ, Liu G, Wu S, Pan A: **Effectiveness of a Workplace-Based, Multicomponent Hypertension Management Program in Real-World Practice: A Propensity-Matched Analysis**. *Hypertension* 2022, **79**(1):230-240.

Table S3. Scenario analysis by simulating the model for 55-year time horizon

Outcome	55-year time horizon		
	Drug treatment	Non-drug treatment	Changes
Discounted QALYs	15.68	14.93	0.75
Discounted cost ($)*	6349.60	7061.74	-712.14
Costs by stage 1 hypertension#	795.97	86.43	709.54
Costs by stage 2 hypertension	1108.59	1392.63	-284.04
Costs by stroke	2056.03	2935.85	-879.82
Costs by MI	254.09	448.02	-193.93
Productivity loss†	2134.92	2198.81	-63.89
Discount ICER	Cost-saving		

MI, myocardial infarction; ICER, incremental cost-effectiveness ratio; and QALY, quality-adjusted life-year.

* US$1.00=6.8974RMB; The per capita GDP of China in 2020 was reported to be $10438.66 from the China Health Statistics Yearbook report.

# Costs for stage 1 hypertension in drug treatment group included costs for hypertension screening or management and costs for antihypertensive drugs, whereas costs for stage 1 hypertension in non-drug treatment group only included costs for hypertension screening or management.

† Productivity losses were only considered in the first 10 years of the microsimulation.

Table S4. Scenario analysis by excluding cost of productivity loss

Outcome	8-year time horizon			15-year time horizon		
	Drug treatment	Non-drug treatment	Changes	Drug treatment	Non-drug treatment	Changes
Discounted QALYs	5.92	5.80	0.12	9.36	9.07	0.29
Discounted cost ($)*	876.81	800.32	74.49	1600.10	1724.66	-124.55
Costs by stage 1 hypertension#	552.54	80.52	472.02	709.74	85.87	623.87
Costs by stage 2 hypertension	215.95	414.38	-198.43	493.39	788.80	-295.41
Costs by stroke	89.08	237.99	-148.91	337.68	701.24	-363.56
Costs by MI	19.24	67.43	-48.19	59.30	148.75	-89.45
Discount ICER	$678.78/QALY			Cost-saving		

MI, myocardial infarction; ICER, incremental cost-effectiveness ratio; and QALY, quality-adjusted life-year.

* US$1.00=6.8974RMB; The per capita GDP of China in 2020 was reported to be $10438.66 from the China Health Statistics Yearbook report.

# Costs for stage 1 hypertension in drug treatment group included costs for hypertension screening or management and costs for antihypertensive drugs, whereas costs for stage 1 hypertension in non-drug treatment group only included costs for hypertension screening or management.

Table S5. Tornado text report (ICER), drug treatment versus non-drug treatment

Variable Name	Variable Low	Variable High	Low	High
Cost for productivity in non-drug treatment group	206.53	275.38	-2552.69	1249.41
RR reduction for mortality in stage 1 hypertension patients	0.1	0.02	-2336.53	-702.82
RR reduction for stroke in stage 1 hypertension patients	0.32	0.15	-528.40	68.44
Cost for antihypertensive drug	66.69	88.92	-922.41	-380.87
Cost for post-stroke	1144.23	1525.64	-854.46	-448.82
Discount rate	0	0.05	-811.69	-535.75
Cost for stroke	2437.16	3249.55	-763.13	-540.15
Utility of hypertension	0.79	0.9	-816.74	-596.80
RR reduction for MI in stage 1 hypertension patients	0.12	0.02	-633.10	-448.91
RR reduction for hypertension progression	0.3	0.39	-731.10	-574.82
Progression to stage 2 hypertension in non-drug treatment	0.2172	0.2408	-696.24	-569.91
Utility of post-stroke	0.46	0.65	-716.40	-591.84
Cost for MI	3533.12	4710.83	-706.28	-597.00
All-cause mortality in non-drug treatment group	0.0037	0.0041	-689.64	-616.31
Incidence of stroke in non-drug treatment group	0.0026	0.0029	-687.50	-615.34
Stroke incidence in patients with stage 2 hypertension	0.0077	0.0081	-686.05	-616.78
Cost for post-MI	321.19	428.26	-674.34	-628.94
Mortality in patients with stage 2 hypertension	0.0082	0.0089	-686.87	-642.02
Incidence of MI in non-drug treatment group	0.0011	0.0013	-670.00	-633.26
Utility of post-MI	0.67	0.88	-660.04	-623.87
Utility of stroke	0.46	0.63	-672.88	-636.78
Cost for hypertension screening	21.53	28.71	-664.83	-638.45
MI incidence in patients with stage 2 hypertension	0.0021	0.0023	-664.46	-638.79
Recurrent MI in non-drug treatment group	0.0237	0.0364	-661.60	-645.12
Recurrent MI in stage 2 hypertension patients	0.0362	0.0449	-660.69	-644.35
Recurrent stroke in stage 2 hypertension patients	0.0491	0.0551	-660.01	-644.26

Utility of MI	0.5	0.76	-655.59	-643.87
Recurrent stroke in non-drug treatment group	0.0225	0.0311	-656.99	-647.74
RR reduction for stroke in stage 2 hypertension patients	0.28	0.14	-650.38	-643.52
RR reduction for post-stroke in stage 2 hypertension patients	0.19	0.26	-654.01	-649.29
RR reduction for post-MI in stage 2 hypertension patients	0.2	0.32	-653.95	-649.73
RR reduction for mortality in stage 2 hypertension patients	0.25	0.13	-656.34	-652.29
Mortality after stroke in stage 2 hypertension patients	0.0012	0.0014	-653.01	-650.27
RR reduction for MI in stage 2 hypertension patients	0.04	0.14	-652.15	-651.13
Mortality after stroke in non-drug treatment group	0.0002	0.0003	-651.97	-650.97
RR reduction for post-stroke in stage 1 hypertension patients	0.16	0.14	-651.58	-650.76
Mortality after MI in stage 2 hypertension patients	0.0003	0.0004	-651.75	-651.53
Mortality after MI in non-drug treatment group	0.0001	0.0002	-651.73	-651.55
Stroke incidence after MI in stage 2 hypertension patients	0.00006	0.00009	-651.77	-651.60
RR reduction for post-MI in stage 1 hypertension patients	0.18	0.27	-651.68	-651.59
Stroke incidence after MI in non-drug treatment group	0.00001	0.00002	-651.69	-651.63
MI incidence after stroke in stage 2 hypertension patients	0.00008	0.00013	-651.67	-651.62
MI incidence after stroke in non-drug treatment group	0.00001	0.00003	-651.64	-651.62

CVD indicates cardiovascular disease; MI, myocardial infarction; and ICER, incremental cost-effectiveness ratio.

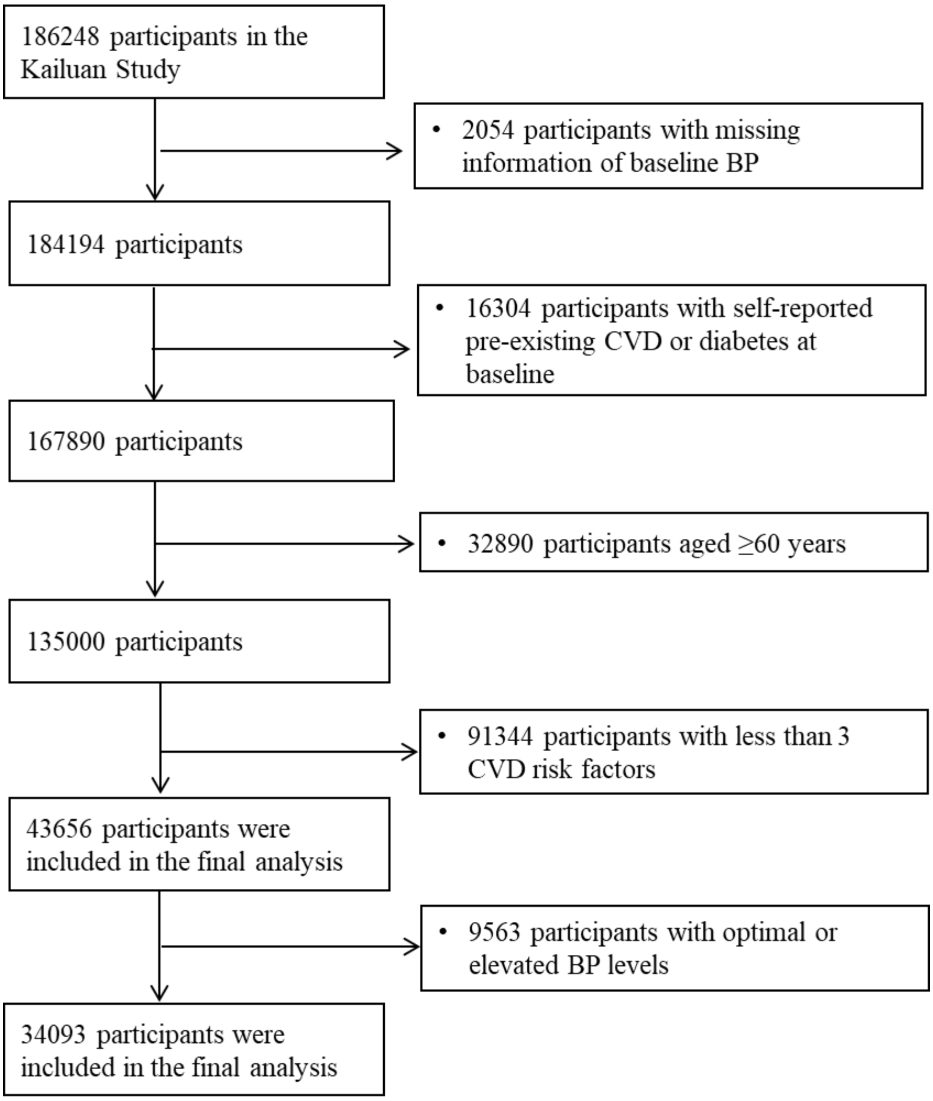

Figure S1. Participant flowchart.

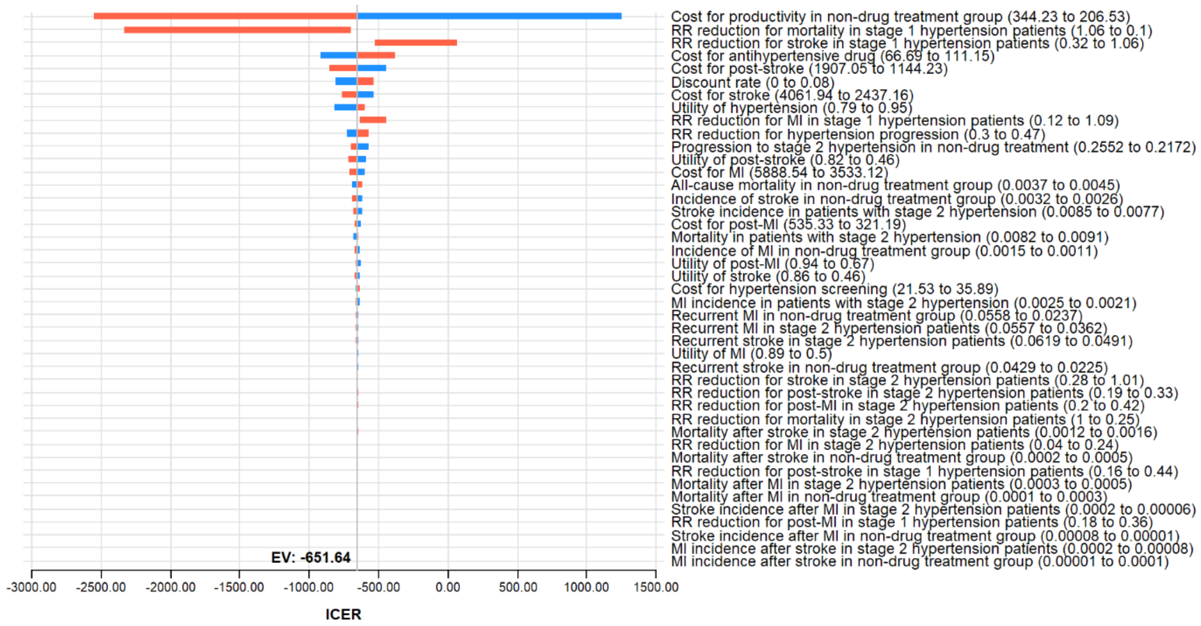

Figure S2. Sensitivity analysis of model variables. For the top parameter Capacity, the incremental value increases as the parameter increases with the blue bar section representing the parameter range from the low uncertainty value to the base case, while the red bar section represents the parameter range from the base case to the high uncertainty value.

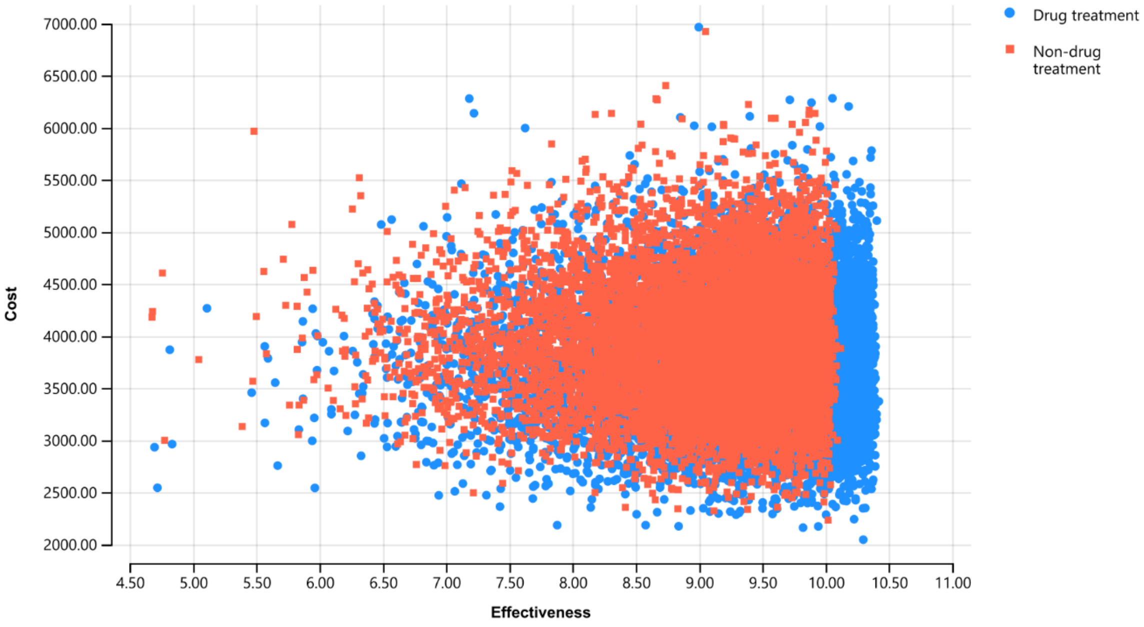

Figure S3. The cost-effectiveness scatter plots for drug treatment versus non-drug treatment
